# Effects of 𝛽-Hydroxy-𝛽-methylbutyrate-free Acid Supplementation on Strength, Power and Hormonal Adaptations Following Resistance Training

**DOI:** 10.3390/nu9121316

**Published:** 2017-12-02

**Authors:** Abbas Asadi, Hamid Arazi, Katsuhiko Suzuki

**Affiliations:** 1 Department of Exercise Physiology, Faculty of Sport Sciences, University of Guilan, Rasht 4199613776, Iran; Abbas_asadi1175@yahoo.com (A.A.); hamidarazi@yahoo.com (H.A.); 2 Faculty of Sport Sciences, Waseda University, 1-104 Totsukamachi, Shinjuku-ku, Tokyo 169-8050, Japan

**Keywords:** HMB free acid, supplementation, muscle mass, sports nutrition

## Abstract

Background: β-Hydroxy-β-methylbutyrate-free acid (HMB-FA) has been ingested prior to exercise to reduce muscle damage, however the effects of HMB-FA supplementation on hormonal, strength and power adaptation are unclear. Methods: Sixteen healthy men were matched and randomized into two groups and performed six-week resistance training while supplementing with either HMB-FA or placebo (3 g per day). The subjects were evaluated for 1 repetition maximum (1RM) bench press and leg press and vertical jump (VJ) prior to and after training intervention. In addition, blood samples were obtained before and after resistance training to evaluate resting growth hormone (GH), insulin like growth factor 1 (IGF-1), testosterone (TEST), cortisol (CORT), and adrenocorticotropic hormone (ACTH) responses. The HMB-FA supplementation group showed greater gains compared with the placebo group in peak power (effect size ES = 0.26 vs. 0.01) and 1RM leg press (ES = 1.52 vs. 0.96). In addition, the HMB-FA supplementation group indicated greater decrements in ACTH and CORT responses to training in comparison to the placebo group (*p* < 0.05). Likewise, in GH (ES = 1.41 vs. 0.12) and IGF-1 (ES = 0.83 vs. 0.41), the HMB-FA indicated greater training effects when compared with the placebo group. Conclusions: These findings provide further support for the potential anabolic benefits associated with HMB-FA supplementation.

## 1. Introduction

Resistance training is one of the best training modalities to enhance muscle strength, power and hypertrophy [1]. Activation of muscle fibers to produce maximal force needs some events including elevation of protein synthesis, activation of satellite cells, muscle cell signaling pathways and hormone responses [1,2]. In fact, these events can promote capability of force production and strength development [3]. In particular, the ingestion of essential amino acids provides increases in strength and power performance following resistance training [3,4,5]. Therefore, amino acid supplementation can promote adaptations to resistance training via several mechanisms (e.g., promoting protein synthesis, attenuating muscle damage and hormone adaptations) [6]. Because of the critical role of amino acids, recent studies have investigated the efficacy of β-Hydroxy-β-methylbutyrate (HMB) supplementation for promoting resistance exercise adaptations [7,8]. HMB is a metabolite of the amino acid leucine, a potent stimulus of translation initiation and protein synthesis [8], and several studies indicated that HMB could enhance muscle size and strength performance [7,9], elevate anabolic hormones [10] and attenuate catabolic hormones [11] following resistance exercise. In addition, HMB increased phosphorylation activity of the mammalian inhibitor target of rapamycin (mTOR) pathway and mTOR initiation factors in the muscle with reduction of the ubiquitin-proteasome proteolysis pathway in humans, resulting in preservation of lean body mass [12,13]. In relation to chronic supplementation with HMB, several research groups in this area reported no change [9,13] and increases [7] in strength and power during a resistance training program.

To date, the majority of researchers utilized supplementation of commercially available calcium salt form of HMB (HMB-Ca) [7,11,12,13] and research studies in this area reported conflicting results when supplemented with HMB-Ca during a resistance training intervention. However, a new free form of HMB (HMB-FA) has been shown to have a more rapid and higher bioavailability compared to HMB-Ca [14,15]. Fuller et al. [14] found that HMB-FA had a 25% greater clearance in the body and faster absorption when compared to HMB-Ca (36 vs. 131 min). These data suggest that acute supplementation with HMB-FA may be more effective than HMB-Ca for reducing muscle damage [16,17,18], inflammatory response [15,16] and anabolic hormones induction [10]. However, to our knowledge, only one study examined the effect of chronic supplementation of HMB-FA on strength and power adaptations following resistance training. Wilson et al. [8] investigated the effects of 12 weeks of HMB-FA supplementation on muscle mass, strength and power in resistance-trained men and found that HMB-FA induced greater gains in comparison to the placebo group. In addition, the creatine phosphokinase and cortisol responses to resistance training were lower for HMB-FA. The greater absorption rate and bioavailability of HMB-FA may provide greater benefits regarding its efficacy as a nutrient supplement used to enhance training adaptations [14,18], thus shortening the length of time needed between supplementation and exercise. This may potentially allow the athlete a shorter recovery time and an increase in performance in subsequent training session.

Regarding the effects of HMB ingestion on hormonal responses to resistance exercise, Tonwsend et al. [10] examined the acute effects of HMB-FA ingestion on the acute endocrine responses to resistance exercise and found that only 1 g HMB-FA ingestion 30 min prior to resistance exercise elevated growth hormone, testosterone and insulin like growth factor 1 (IGF-1) concentrations at post exercise. Although this study explored the acute effects of HMB-FA ingestion on hormonal responses, the chronic effects of HMB-FA supplementation with resistance training on resting hormonal response to resistance training is unclear. Moreover, to the best of our knowledge, there are no previous studies that have examined the influence of HMB-FA supplementation on hormonal adaptations and changes in strength and power performance during six-week resistance training. Therefore, the aim of the present study was to investigate the influence of chronic HMB-FA supplementation on hormonal adaptations and their relationships to muscle strength and power development during the six-week resistance-training period in men.

## 2. Materials and Methods

### 2.1. Participants

Initially, twenty healthy subjects volunteered to participate for the study, but four subjects did not continue training, thus 16 of them completed the whole six-week investigation and testing sessions (Table 1). A priori calculations of statistical power indicated that this sample size was appropriate to satisfy power at or above 80% [19]. The subjects were free from any lower and upper body injuries, and had no history of musculoskeletal, neurological, or orthopedic disorders that might have affected their inclusion to resistance training. Subjects were not permitted to use any nutritional supplements or medications while enrolled in the study. Screening for nutritional, drug and hormonal supplements was accomplished via a health-history questionnaire, completed during participant recruitment. Subjects were informed about the potential risks and discomforts associated with the investigation, and all subjects gave their written, informed consent to participate. The Ethics Committee of the University approved the study (DT-167908).

### 2.2. Experimental Protocol

The present study is a longitudinal research with the placebo-controlled, double-blind, randomized design. Two weeks prior to the initiation of resistance training period, all subjects were familiarized with the testing and training procedures. During this session, subjects’ anthropometric measurements were determined and the subjects completed a familiarization session with equipment, resistance training room and proper strength and power tests. To standardize the training procedures, a one-week orientation took place consisting of two sessions in which the methods and techniques of the training programs were demonstrated. Each subject performed at least five familiarization sessions and participated in a preconditioning program before the commencement of the intervention. Strength and power measurements were taken one week before and after intervention, completed in two non-consecutive days and were always administered in the same order, at the same time of the day and by the same investigators. On Day 1, height, weight and countermovement jump were measured. On Day 2, 1 repetition maximum (1RM) of leg press and bench press were measured. Forty-eight hours prior to and after completing the training period, blood samples were taken to determine resting hormone levels.

### 2.3. Testing Procedures

All performance testing sessions were conducted at the same time of day (i.e., afternoon) to account for diurnal performance effects that have typically been noted when assessing markers of strength and power by Certified Strength and Conditioning Specialists. Before testing, each subject performed ten minutes of standard warm-up.

#### 2.3.1. Anthropometric Measures

Subject’s height was measured using a wall-mounted stadiometer (Seca 222, Terre Haute, IN, USA) recorded to the nearest centimeter. Weight was measured to the nearest 0.1 kg using a medical scale (Tanita, BC-418MA, Tokyo, Japan). The body mass index (BMI) was determined by dividing body mass by the square height of the subject (kilograms per square meters).

#### 2.3.2. Strength and Power Measures

For the upper and lower body strength, bench and leg press exercises were measured with a series of 1RM tests using free weights (Nebula Fitness, Inc., Versailles, OH, USA) as previously described [20]. Briefly, participants performed a warm-up on a cycle ergometer followed by light dynamic stretching. In the next step, participants performed 8–10 repetitions at 50% of estimated 1RM, followed by another set of 3–5 repetitions at 85% of 1RM. Three to four maximal trials separated by 2–3 min of rest were used to determine individual 1RM for each resistance exercise. For the countermovement jumps, participants executed maximal effort jumps without arms akimbo. The participants were instructed to flex their knees until 90° and completed three maximal countermovement vertical jumps each separated by a 30 s rest period, according to previously established methods [21]. The participants were instructed to maximize jump height. To estimate vertical jump peak power (W), a previously established testing protocol and equation (W = 0.67 × jump height (cm) + 45.3 × body mass (kg) − 2.055) was used [22].

#### 2.3.3. Blood Measures and Analysis

Blood samples were drawn (10 mL) from the antecubital vein into plain evacuated test tubes at rest between 8:00 and 9:00 a.m. before and after resistance training to evaluate resting growth hormone (GH), insulin-like growth factor 1 (IGF-1), testosterone (TEST), cortisol (CORT), adrenocorticotropic hormone (ACTH), creatinine and urea responses to the training period. The blood was allowed to clot at room temperature for 30 min and centrifuged at 1500× *g* for 10 min. The serum layer was removed and frozen at –80 °C in multiple aliquots for further analyses. Resting blood samples were drawn at 48 h before the start of training (pre) and 48 h after the last training session (post), after a 10 h fast and after 8 h of sleep to control of the circadian hormonal range. The time of blood collection was chosen based on the previous studies conducted with these procedures for the control of the circadian hormonal range [23]. All analyses were performed by radioimmunoassay (RIA) method with standardized procedures using commercially available kits (Monobind, Inc. Lake Forest, CA, USA). The intra-assay variance was determined based upon a coefficient of variation (CV) <6%. Serum concentrations of creatinine (Cr) and urea were measured using an automated analyzer (Model 747-400, Hitachi, Tokyo, Japan).

### 2.4. HMB-FA Supplementations

The HMB-FA supplement consisted of 1 g of β-Hydroxy-β-methylbutyrate-free acid (BetaTor, Body Attack, GmbH & Co. KG, Waldhofstrabe 19, 25474, Ellerbek, Germany). Each serving of placebo consisted of an equivalent amount of polydextrose. Participants consumed either the supplement or placebo 3 times per day. A serving size of 1 capsule (1 g) at each meal was used. The placebo was indistinguishable in appearance and taste and was provided in the same form as the supplement. The HMB-FA supplement time points on training day were during breakfast, 30 min prior to exercise, and supper. Likewise, on non-training days, the participants were instructed to consume one serving with each meal throughout the day. Participants were required to consume three servings per day were provided with weekly supplies of HMB-FA and placebo. Participants were required to return all used and unused packets at the end of each week. The compliance for the supplement ingestion was 100%.

### 2.5. Diet Control

Participants were prevented to take nutritional supplements or any drugs which promotes performance for at least 6 months before data collection. Participants completed 3-day diet records at pre-training and during the last week of training period. Food diaries were analyzed for energy and macro/micronutrient content and the participants had similar caloric intake regarding 25% protein, 50% carbohydrates and 25% fat. After the first 3-day diet record, but before the onset of the training period, participants received dietary counseling by a registered dietitian to ensure that the participants maintained their habitual dietary habits during the study (Table 2).

### 2.6. Training Program

The resistance training protocol consisted of a 6-week (12 sessions) program. Each session lasted 70–80 min. Ten minutes of standard warm-up (i.e., 5 min of submaximal running and several displacements and stretching exercises for 5 min) was used before the main part of the training session. The resistance exercise program stressed major muscle groups and included the following exercises (or variations of) in each session: leg presses, knee flexions, knee extensions, lat pull downs, bench presses, shoulder presses, cable biceps curl and triceps push-downs which participants performed 3 sets of 8–12 repetitions at 75–85% of 1RM with 2 and 3 min rest intervals between sets and exercises, respectively. Weight was increased systematically if the prescribed amount of repetitions were completed. All participants in the present study were required to complete all training and each training session was monitored by Certified Strength and Conditioning Specialists to ensure that all training exercise sessions were performed correctly with the appropriate loads and rest intervals. Make up sessions were allowed if subjects missed a regularly scheduled training session; therefore, participants’ compliance in this study was 100% for the chronic training protocol.

### 2.7. Statistical Analyses

Pre- and post-values for the dependent variables were analyzed to determine if the distributions were normal using the Shapiro-Wilk Normality test. A 2 (group) by 2 (time) repeated measures ANOVA was utilized to analyze changes in the dependent variables measured at baseline and after 6 weeks of supplementation and resistance training. The calculation of effect size was used to examine the magnitude of any treatment effect. The effect size statistics was considered: trivial, <0.20; small, 0.20–0.50; moderate, 0.5–0.80; large, 0.8–1.30; or very large >1.30 [24]. The effect size is reported in conjunction with the 95% confidence interval (CI) for all analyzed measures. Pearson product-moment correlations coefficient was used to determine relationships between performance variables and hormonal changes (*n* = 16). Statistical significance was set at *p* ≤ 0.05 for these analyses. All values are presented as mean ± standard deviation (SD). All analyses were conducted using SPSS version 21.0 (SPSS Inc., Chicago, IL, USA).

## 3. Results

Before training, no significant differences were observed between groups in height, body mass and BMI (*p* > 0.05). After training, no significant changes in the body mass were observed for the placebo supplementation group (from 78.1 ± 13.4 to 78.9 ± 13.3 kg, *p* > 0.05), while the HMB-FA supplementation group indicated significant changes (from 79.2 ± 13.5 to 82.8 ± 13.6 kg, *p* ≤ 0.05).

### 3.1. Strength and Power Measures

**Figure 1:**
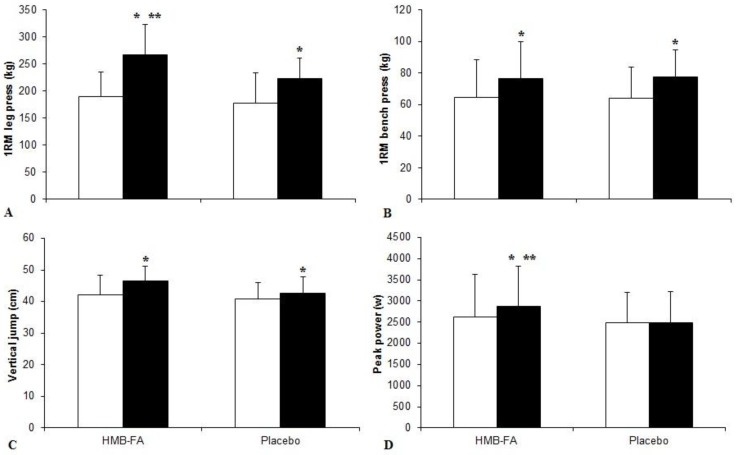
Changes in strength and power following six-week training intervention (mean ± SD). (**A**): 1RM leg press; (**B**)**:** 1RM bench press; (**C**)**:** vertical jump; (**D**)**:** peak power. *: denotes significant differences between baseline and post training values (*p* ≤ 0.05); **: denotes significant differences between the HMB-FA and placebo supplementation groups at post training (*p* ≤ 0.05). (□ pre-test; ■ post-test).

Changes in 1RM of leg and bench presses are presented in Figure 1A,B. The 1RM of leg press was increased significantly in both HMB-FA supplementation (from 189.2 ± 46.5 to 267 ± 55.6 kg; ES = 1.52, 95% CI = −0.34 to 2.53, *p* ≤ 0.05) and placebo supplementation (from 178.3 ± 54.8 to 223.5 ± 37.3 kg; ES = 0.96, 95% CI = −0.12 to 1.94, *p* ≤ 0.05) groups after six weeks of training (*p* ≤ 0.05). Compared with placebo supplementation, the HMB-FA group showed significant group by time interaction, which indicated significant differences with placebo supplementation group in 1RM of leg press (*p* ≤ 0.05). The 1RM of bench press was increased significantly in both HMB-FA supplementation (from 64.3 ± 24.1 to 76.3 ± 23.6 kg; ES = 0.51, 95% CI = −0.52 to 1.47) and placebo supplementation (from 64.2 ± 19.5 to 77.6 ± 16.8 kg; ES = 0.74, 95% CI = −0.31 to 1.71) groups after six weeks of training (*p* ≤ 0.05), without differences between groups (*p* ≥ 0.05).

Both groups demonstrated significant increases (HMB-FA: from 42.2 ± 6 to 46.5 ± 4.8 cm; ES = 0.79, 95% CI = −0.27 to 1.76; placebo: from 40.7 ± 5.2 to 42.5 ± 5.2 cm; ES = 0.35, 95% CI = −0.66 to 1.31; *p* ≤ 0.05) in the vertical jump test (Figure 1C). Likewise, the HMB-FA supplementation group indicated moderate training effects (ES = 0.79), while placebo supplementation group showed small training effects (ES = 0.35) and these differences were not statistically significant (*p* > 0.05). Additionally, the HMB-FA supplementation induced moderate significant increases (from 2625.0 ± 1007.9 to 2874.4 ± 940.8 w; ES = 0.26, 95% CI = −0.74 to 1.23; *p* ≤ 0.05) in the peak power output achieved during the vertical jump, while the placebo group did not show any significant changes in the peak power after six weeks of training (from 2481.8 ± 713.6 to 2492.1 ± 723.1 w; ES = 0.01, 95% CI = −0.97 to 0.99; *p* ≥ 0.05). Compared with placebo supplementation, the HMB-FA group showed significant group by time interaction, which indicated greater significant training effects in HMB-FA compared to placebo supplementation group in the peak power output achieved during the vertical jump (*p* ≤ 0.05) (Figure 1D).

### 3.2. Hormonal Measures

Changes in hormonal measures before and after the training intervention are presented in Table 3. After the six-week resistance training intervention both the HMB-FA and placebo supplementation groups displayed a significant (*p* ≤ 0.05) increase in the GH concentration. No group × time interactive effects for GH was observed, but there was a significant and very large increase for the HMB-FA supplementation group (ES = 1.41) and a trivial increase for the placebo supplementation group (ES = 0.12) in GH concentration. Both the HMB-FA and placebo supplementation groups displayed significant increase in IGF-1 concentrations after the six-week training intervention (*p* ≤ 0.05). The HMB-FA and placebo supplementation groups indicated large (ES = 0.83) to small (ES = 0.41) enhancements after resistance training, respectively. No group × time interactive effects for IGF-1 were observed (*p* ≥ 0.05). There was no significant main effect for group or time and group × time interactive effects for TEST concentration (*p* ≥ 0.05). However, HMB-FA and placebo supplementation groups exhibited moderate (ES = 0.57) to trivial (ES = 0.08) enhancements after six-week training intervention, respectively. HMB-FA supplementation group showed a statistically significant (*p* ≤ 0.05) decrease in CORT and ACTH concentrations, with a very large (ES = −1.89; −1.76, respectively) meaningful effect. Placebo supplementation group also showed no significant (*p* ≥ 0.05) change in the CORT concentration. For ACTH, placebo supplementation group revealed significant (*p* ≤ 0.05) decrease, with large (ES = −1.04) meaningful effect. Compared with placebo, the HMB-FA supplementation group showed a significantly (*p* ≤ 0.05) greater changes in CORT and ACTH concentrations. No group × time interactive effects or group and time effects were observed for Cr and urea (*p* ≥ 0.05). However, placebo supplementation group showed greater increases in Cr (ES = 0.59 vs. 0.01) and urea (ES = 0.8 vs. 0.61) in response to six-week training intervention when compared with the HMB-FA supplementation group.

As shown in Table 4, there was significant correlations between post-training IGF-1 with 1RM leg press (r = 0.649, *p* = 0.006) and 1RM bench press (r = 0.623, *p* = 0.01), whereas there were no significant correlations between other variables (*p* ≥ 0.05).

## 4. Discussion

The primary findings of this study were that HMB-FA supplementation for six weeks induced greater gains in strength (1RM leg press), and vertical jump peak power in comparison to the placebo-supplemented group. Both experimental groups elicited significant increases in GH and IGF-1 concentrations, whereas the HMB-FA supplementation group indicated greater meaningful changes (ES = 1.41 vs. 0.12 in GH, and ES = 0.83 vs. 0.41 in IGF-1) after six weeks of training intervention. Finally, supplementation with HMB-FA reduced the rise in CORT and ACTH following training over the six-week study compared with the placebo-supplemented group.

Regarding critical roles of muscle strength and power to succeed in sport [25], many studies examined the effects of resistance training on strength and power performance and reported that resistance training can improve strength performance and enhance the ability to express high power outputs during jumping movements [21,25,26], which are in line with our findings.

In relation to HMB supplementation, the findings of the present study indicated that HMB-FA supplementation not only increased the strength (i.e., 1RM leg press) and the power performance, but also these improvements were greater in comparison to the placebo supplementation group (except 1RM bench press and vertical jump). In accordance with our results, Wilson et al. [8] found that 12 weeks of HMB-FA supplementation induced meaningful gains in the strength and power performance when compared to placebo supplementation. The greater enhancements in muscular strength and power observed in the HMB-FA supplementation group may be indicative of greater force-related adaptations via HMB-FA supplementation [27]. Particularly, HMB-FA supplementation has been shown to increase the cross-sectional areas of the muscle fibers, as well as lean body mass [8], resulting in greater strength and power gains after training. In contrast, some studies did not find any significant effects of HMB supplementation (i.e., HMB-Ca) on strength gains [9,13]. It seems that more rapid and higher bioavailability of HMB-FA form induced more changes in lean body mass by stimulating protein synthesis resulting in greater gains in strength and power.

With review of hormonal changes (Table 2), these results suggest that HMB-FA may affect growth hormone/ insulin like growth factor 1 (GH/IGF-1) axis signaling; however, the effect on skeletal muscle protein synthesis requires more investigation. It is possible that the GH/IGF-1 axis signaling could require a large change in plasma HMB (i.e., HMB-FA) levels and it appears that chronic supplementation with free acid HMB form induced meaningful effects on GH/IGF-1 axis (very large to large training effects, Table 2) resulting in body mass increase and strength gain [27].

Another possible mechanism for the discrepancy could be due to the measurement technique. Previous studies, using compound, sport-specific movements such as the squat, bench press, and vertical jump, have found robust changes in the strength and power following HMB supplementation [8,28]. In contrast, other studies have found small treatment effects using non-specific, isolated movements such as the leg extension and preacher curl [29,30]. Taken together, these results suggest that HMB supplementation affects strength and power gains; however, this certainly merits further investigation.

In relation to anabolic hormone adaptations to training, we found that six weeks of resistance training induced meaningful gains in GH and IGF-1 concentrations for both the HMB-FA and placebo groups, but these improvements were greater for the HMB-FA supplementation group (ES = 1.41 vs. 0.12 in GH, and ES = 0.83 vs. 0.41 in IGF-1). For TEST, neither the HMB-FA nor placebo supplementation group showed statistically significant improvement, but the HMB-FA supplementation group indicated greater training effects (ES = 0.57 vs. 0.08) compared to the placebo supplementation group. Changes in the resting GH, IGF-1 and TEST concentrations during resistance training have been inconsistent in men. On the other hand, elevated resting GH, IGF-1 and TEST concentrations have been reported in some studies [4,7,31], whereas several studies have shown no differences [32,33] or reductions [34]. To our knowledge, this was the first study that examined the effects of chronic HMB-FA supplementation on hormonal adaptations after resistance training; however, in an earlier study by Townsend et al. [10], enhancement of GH and IGF-1 occurred only by acute ingestion of 1 g HMB-FA prior to a session of resistance exercise. Regarding chronic HMB supplementation and resistance training, previous studies which have used HMB-Ca supplementation indicated no changes or trivial increases in resting GH, IGF-1 and TEST concentrations [7,13]. In contrast, the results of the present study indicated that HMB-FA supplementation induced very large and large meaningful effects in GH and IGF-1 concentrations following six weeks of resistance training, without statistically significant effects in TEST concentration. Whereas HMB-Ca induces modest or trivial elevations in GH and IGF-1, higher absorption and greater bioavailability of leucine metabolites in new form (i.e., HMB-FA) demonstrated to stimulate larger increases in GH and IGF-1 concentrations [10,35].

CORT and ACTH are two main hormones to increase catabolic status of the body. The catabolic functions of CORT and ACTH have greater effects in type II muscle fibers [4,31]. In the peripheral tissues, CORT stimulates protein degradation and decrease in protein synthesis in muscle cells, resulting in greater release of amino acids into the circulation [5]. In addition, resting levels of CORT and ACTH concentrations generally reflect a long-term training stress [8,31]. In this study, we found that six weeks of resistance training induced decrements in resting ACTH, but did not change the CORT concentration. The HMB-FA supplementation group showed very large and large meaningful effects in CORT (ES = −1.89) and ACTH (ES = −1.04,) concentrations after a six-week training intervention. Elevated resting CORT and ACTH concentrations have been reported in a few studies [36], although other studies have shown reduction or no differences [32,33,34]. The decrease in the resting concentrations of CORT throughout the training program with significant reduction of ACTH concentrations indicates that the ACTH receptors in the adrenal gland may have been “downregulated” [33]. With training, reductions in CORT response to resistance training may provide protein accretion via reduced degradation in the muscle fibers. ACTH has been shown to mediate the secretion of CORT from the adrenal cortex and it has been established that CORT secretion and clearance are coupled [32,33]. In relation to HMB supplementation (i.e., HMB-Ca), some studies indicated decrement [7,28], while one study showed no differences in CORT and ACTH concentration following resistance training [11]. The possible explanations for the discrepancy in results observed among studies about the effect of HMB supplementation could be due to differences in experimental design (e.g., dietary controls, type and intensity of training, subject training status, duration of experiment, etc.), and methods applied (e.g., supplement formulations investigated) among studies. Regarding reduction of CORT and ACTH concentrations in response to training, previous studies have reported that HMB supplementation (i.e., HMB-Ca) have trivial or small meaningful effects [7,11], whereas, in this study, we found that HMB-FA supplementation not only blunted the rise of ACTH and CORT, but also decreased very large meaningful effects (CORT: ES = −1.89 and ACTH: ES = −1.76). It appears that HMB-FA supplementation has anti-catabolic effects and may reduce skeletal muscle proteolysis [13]. Di Pasquale [37] has postulated that HMB may regulate protein metabolism either by hormonal receptor effects or by modulating the enzymes responsible for muscle tissue breakdown. On the other hand, HMB has been shown to decrease muscle protein degradation in humans via decrease of proteasome expression [10,13] which have been shown by reducing CORT and ACTH concentrations without changes in Cr and urea. In this study, serum Cr and urea did not change in HMB-FA supplementation group that is in line with anti-catabolic and anti-proteolysis effects of HMB-FA.

In the present study, we determined the relationship between IGF-1 with 1RM leg press and bench press, but no correlations were found between other hormones and performance variables. These results revealed that muscular strength could be a predictor of the IGF-1 levels in humans. On the other hand, greater strength level could be in line with greater IGF-1 levels at post-training; however, these relationships need to be examined in more studies.

## 5. Conclusions

In summary, the results of this six-week study demonstrated the efficacy of HMB-FA supplementation on strength and power performance. The use of these supplements appears to provide greater changes compared with placebo supplementation. HMB-FA supplementation altered resting hormonal concentrations, and the results appear to be the first examination suggesting that HMB-FA supplementation for six weeks can promote a significantly greater post-training increase in GH and IGF-1. To reduce catabolic hormones, HMB-FA induced very large, meaningful effects in CORT and ACTH concentrations after six weeks of training intervention. It could be concluded that six weeks of HMB-FA supplementations induced meaningful increases in anabolic hormones with reduction of catabolic hormones and remarkable gains in strength and power performance.

## Figures and Tables

**Table 1:** Subject characteristics (mean ± SD).

	HMB-FA (*n* = 8)	Placebo (*n* = 8)
Age (year)	21.5 ± 0.5	21.3 ± 0.9
Height (cm)	181.7 ± 4.1	179.5 ± 4.5
Body mass (kg)	79.2 ± 13.0	78.9 ± 13.3
BMI (kg/m^2^)	23.9 ± 3.2	24.4 ± 4

BMI: Body mass index; HMB-FA: β-Hydroxy-β-methylbutyrate-free acid.

**Table 2:** Dietary intake assessed for the HMB-FA and placebo supplementation groups.

		HMB-FA (*n* = 8)	Placebo (*n* = 8)
Energy intake (kcal)	Before	2532 ± 210	2421 ± 176
	After	2891 ± 198	2782 ± 215
Carbohydrate (g)	Before	260 ± 23	259 ± 29
	After	282 ± 31	278 ± 41
Fat (g)	Before	75 ± 11	79 ± 13
	After	84 ± 14	81 ± 17
Protein (g)	Before	98 ± 19	93 ± 21
	After	119 ± 22	115 ± 34
Vitamin E (mg)	Before	8.9 ± 1.0	9.0 ± 2.0
	After	10.0 ± 2.0	9.5 ± 1.6
Vitamin C (mg)	Before	65 ± 22	67 ± 17
	After	77 ± 18	78 ± 15

Before and after the training period. Data are presented as mean ± SD. HMB-FA: β-Hydroxy-β-methylbutyrate-free acid.

**Table 3:** Changes in hormonal responses to six-week training intervention (mean ± SD).

	HMB-FA (*n* = 8)		Placebo (*n* = 8)		Significance
**GH (ng/mL)**					
Pre	0.10 ± 0.03		0.11 ± 0.04		G = 0.98
Post	0.19 ± 0.09 *		0.17 ± 0.07 *		T = 0.02
Effect Size	1.41 (−0.25, 2.42)	Very large	0.12 (−0.87, 1.10)	Trivial	G × T= 0.51
**IGF-1 (ng/mL)**					
Pre	256.2 ± 28.5		250.0 ± 36.9		G = 0.55
Post	284.0 ± 37.9 *		266.5 ± 43.6 *		T = 0.05
Effect Size	0.83 (−0.23, 1.80)	Large	0.41 (−0.60, 1.38)	Small	G × T = 0.331
**TEST (ng/mL)**					
Pre	5.47 ± 0.77		5.33 ± 1.10		G = 0.08
Post	6.11 ± 1.40		5.41 ± 0.75		T = 0.33
Effect Size	0.57 (−0.46, 1.53)	Moderate	0.08 (−0.90, 1.38)	Trivial	G × T= 0.53
**CORT (µg/dl)**					
Pre	23.0 ± 4.6		23.9 ± 2.9		G = 0.07
Post	16.6 ± 4.3 *^,^**		24.5 ± 5.4 *		T = 0.02
Effect Size	−1.89 (−2.94, 0.62)	Very large	0.14 (−0.85,1.11)	Trivial	G × T = 0.05
**ACTH (pg/mL)**					
Pre	58.1 ± 10.8		68.8 ± 5.4		G = 0.06
Post	38.1 ± 12.1 *^,^**		60.1 ± 10.5 *		T = 0.002
Effect Size	−1.76 (−2.8, 0.52)	Very large	−1.04 (−2.02, 0.05)	Large	G × T = 0.004
**Cr (mg/dL)**					
Pre	0.98 ± 0.06		1.01 ± 0.09		G = 0.16
Post	0.98 ± 0.09		1.06 ± 0.08		T = 0.16
Effect Size	0.01 (−0.98, 1.00)	Trivial	0.59 (−0.44, 1.55)	Moderate	G × T = 0.27
**Urea (mg/dL)**					
Pre	11.6 ± 1.9		11.6 ± 1.6		G = 0.70
Post	12.8 ± 2.0		13.6 ± 3.2		T = 0.06
Effect Size	0.61 (−0.42, 1.58)	Moderate	0.8 (−0.26, 1.77)	Moderate	G × T = 0.24

*: Denotes significant differences between baseline and post-training values (*p* ≤ 0.05); **: Denotes significant differences between the HMB-FA and placebo supplementation groups at post-training (*p* ≤ 0.05). GH: growth hormone; IGF-1: insulin-like growth factor 1; TEST: testosterone; CORT: cortisol; ACTH: adrenocorticotropic hormone; Cr: creatinine; G: group; T: time.

**Table 4:** Relationships between hormonal measures with strength and power in the post-training intervention (*n* = 16).

	1RM Leg Press (kg)	1RM Bench Press (kg)	Vertical Jump (cm)	Peak Power (w)
GH (ng/mL)	r = 0.242 *p* = 0.366	r = 0.031 *p* = 0.911	r = 0.262 *p* = 0.328	r = −0.194 *p* = 0.472
IGF-1 (ng/mL)	r = 0.649 *p* = 0.006	r = 0.623 *p* = 0.01	r = 0.272 *p* = 0.307	r = 0.155 *p* = 0.567
TEST (ng/mL)	r = 0.00 *p* = 0.999	r =0. 093 *p* = 0.732	r = 0.001 *p* = 0.997	r = 0.278 *p* = 0.297
CORT (µg/dl)	r =−0.126 *p* = 0.641	r = 0.135 *p* =0.618	r = −0.299 *p* = 0.261	r = −0.094 *p* = 0.730
ACTH (pg/ml)	r = -0.178 *p* = 0.509	r = −0.120 *p* = 0.659	r = −0.330 *p* = 0.211	r = −0.081 *p* = 0.766

GH: growth hormone; IGF-1: insulin-like growth factor 1; TEST: testosterone; CORT: cortisol; ACTH: adrenocorticotropic hormone; 1RM: 1 repetition maximum. Bold denotes significant (*p* ≤ 0.05) correlation.
